# Duloxetine repositioning: Investigation of antifungal and antibiofilm activity against fluconazole-sensitive and resistant *Candida* Spp. strains

**DOI:** 10.1007/s42770-026-02038-z

**Published:** 2026-08-03

**Authors:** Sarah Alves Barbosa, Dávylla Rênnia Saldanha Pinheiro, Leilson Carvalho de Oliveira, Lara Elloyse de Almeida Moreira, Vitória Pessoa de Farias Cabral, Daniel Sampaio Rodrigues, Beatriz Oliveira de Souza, Hélio Vitoriano Nobre Júnior, Livia Gurgel Do Amaral Valente Sa, Bruno Coêlho Cavalcanti, Islay Lima Magalhães, José Roberto de Oliveira Ferreira, Manoel Odorico de Moraes, João Batista de Andrade Neto, Cecília Rocha da Silva

**Affiliations:** 1 Department of Clinical and Toxicological Analyses, Faculty of Pharmacy, Laboratory of Bioprospecting of Antimicrobial Molecules (LABIMAN), Federal University of Ceará, Fortaleza, CE Brazil; 2 Nucleus for Drug Research and Development (NPDM), Federal University of Ceará, Fortaleza, CE Brazil; 3 Christus University (UNICHRISTUS), Fortaleza, CE Brazil; 4 Department of Physiology and Pharmacology, Faculty of Medicine, Federal University of Ceará, Fortaleza, CE Brazil; 5 School of Medical Sciences, State University of Health Sciences of Alagoas (UNCISAL), Maceió, AL Brazil

**Keywords:** Candida, Duloxetine, Drug repurposing, Resistance, Biofilm

## Abstract

The increase in fungal infections, the limited therapeutic arsenal, and the emergence of resistance pose a global health problem. *Candida* spp. stand out as opportunistic pathogens that cause superficial and invasive diseases. Thus, the search for new therapeutic alternatives, such as drug repositioning, is necessary. Duloxetine (DUL), a serotonin-norepinephrine reuptake inhibitor antidepressant used to treat depression, has demonstrated antifungal activity and potentiates the effects of conventional antifungals in vitro. The objective of this study is to evaluate the in vitro activity of duloxetine (DUL) against resistant *Candida* spp. strains. To this end, the minimum inhibitory concentration (MIC) of DUL alone and in combination with conventional antifungals was determined in order to evaluate the type of interaction between them, as well as the minimum fungicidal concentration (MFC). The activity of DUL against mature and developing biofilms was evaluated, in addition to the possible antifungal mechanism of action. DUL showed MICs of 16 to 128 µg/mL with a fungicidal action profile, and when combined there was synergistic interaction with amphotericin B (AMB). In biofilms, DUL exhibited antibiofilm activity and enhanced AMB activity against developing, but not mature, biofilms. Mechanism of action analysis showed that DUL promoted oxidative stress, evidenced by increased reactive oxygen species (ROS) production, reduced GSH levels, mitochondrial depolarization, and phosphatidylserine externalization, suggesting the activation of the apoptotic pathway as a possible mechanism of cell death. The pro-oxidant effects of DUL were evident in *C. albicans* strains deficient in antioxidant defenses (*cap1Δ* and *gpx3Δ*) compared to the wild-type strain. DUL exhibited antibiofilm activity and enhanced AMB activity against developing, but not mature, biofilms.

**Supplementary Information:**

The online version contains supplementary material available at 10.1007/s42770-026-02038-z.

## Introduction

Fungal infections are a major public health problem, responsible for approximately 6,5 million deaths annually worldwide. They have a broad clinical spectrum, ranging from superficial to invasive manifestations, mainly in immunocompromised patients [1, 2].

*Candida* spp. stand out for inclusion on the WHO’s list of critical pathogens [3]. This prioritization pertains to fungal pathogens for which there are challenges of drug resistance or other treatment problems [3]. They are opportunistic and are present in commensal form in various regions of the body, mainly in the mucous membranes, in addition to being associated with systemic infections that cause about 60% mortality in some patient groups [4, 5].

Although *C. albicans* is the most prevalent species, there are increases in the species *C. glabrata*, *C. tropicalis*, *C. parapsilosis*, *C. dubliniensis*, *C. guilliermondii*, and *C. krusei*, known as non-*albicans Candida* (NAC) [2, 4, 6]. *C. auris* is of great clinical relevance due to the increase in cases and because it presents simultaneous resistance to the main classes of antifungals [7]. Biofilm formation is an important virulence factor due to high resistance to antifungals and the host’s immunological mechanisms, which makes treatment difficult by favoring the persistence of infections [4].

There are four main classes of antifungals, namely polyenes, azoles, echinocandins, and pyrimidine analogs [1]. There are reports of resistance to all these drugs, either intrinsic or acquired, making treatment more difficult and time-consuming [8]. A major cause of this resistance is the misuse and/or overly extensive use of antifungals, making strains resistant and increasing mortality rates [9].

Given the limited arsenal of antifungals associated with resistance, it is necessary to develop viable treatment strategies. Among these strategies is drug repositioning, which reduces time and cost in comparison with the development of a new drug [10]. The search for pharmacological combinations with synergism is another relevant approach, capable of enhancing therapeutic efficacy and expanding treatment options.

Duloxetine (DUL) is a serotonin and norepinephrine reuptake inhibitor (SNRI), widely used for the treatment of major depressive disorder (MDD), generalized anxiety disorder (GAD), fibromyalgia, and diabetic peripheral neuropathy [11, 12]. DUL has already been used in drug repositioning due to its action on pain and fibromyalgia [10]. Its antifungal potential has been studied against *Cryptococcus* spp. and *Candida* genus, however, its effects on biofilms and its antifungal mechanism of action have not yet been explored.

Thus, the objective of this study was to evaluate the antifungal activity of DUL against *Candida* strains, both in planktonic cells and in biofilms, isolated and in combination with the antifungals fluconazole (FLC), itraconazole (ITC), and AMB, and to investigate its mechanism of action more comprehensively.

## Materials and methods

### Drugs and microorganisms

Fourteen isolates of *Candida* spp. were used, while *C. parapsilosis* ATCC 22,019 and *C. krusei* ATCC 6258 were used as controls. The strains were obtained from the mycotheca of the Laboratory of Bioprospecting Antimicrobial Molecules of Federal University of Ceará (LABIMAN/UFC).

FLC, ITC and AMB were obtained from Sigma-Aldrich (St. Louis, MO, USA) and DUL was obtained from Fragon (São Paulo, Brazil). The DUL, ITC and AMB were solubilized in dimethyl sulfoxide (DMSO) at a final concentration of 2.5%, while FLC was dissolved in distilled water.

### Determination of the minimum inhibitory concentration (MIC)

The MIC was determined by broth microdilution according to protocol M27-A3 [13]. The DUL was tested from 1024 to 2 µg/mL, FLC between 64 and 0.125 µg/mL, and ITC and AMB from 16 to 0.0313 µg/mL, according to protocol M27-S4 [14]. The MIC corresponded to the lowest concentration capable of inhibiting 50% of the microorganisms by visual analysis [13]. The cutoff points for sensitivity and resistance to antifungals were evaluated considering document M27-S4 [14].

### Determination of the minimum fungicidal concentration (MFC)

The MFC was defined as the lowest concentration capable of completely inhibiting fungal growth after seeding on Sabouraud agar (Kasvi, Paraná, Brazil). The classification was carried out according to Siddiqui et al., [15], whereby CFM/CIM ratios ≤ 4 are considered fungicidal and those with ratios > 4 are fungistatic.

### Combination of DUL with antifungals

DUL and antifungals were combined by the checkerboard technique [16], using the MIC value of these drugs. The fractional inhibitory concentration index (FICI) was calculated according to Jorge et al. [17], classified as: Synergistic: FICI ≤ 0.5; Additive: 0.5 < FICI ≤ 1.0; Indifferent: 1 < FICI ≤ 4.0 and Antagonistic: FICI > 4.0.

### Evaluation of cytotoxicity in peripheral blood mononuclear cells (PBMCs)

PBMCs were obtained from healthy donors by density gradient separation with Histopaque-1077. The protocol was approved by the Research Ethics Committee on Human Beings of Federal University of Ceará (protocol number 161/2014).

Cells were cultured in RPMI 1640 supplemented with 10% fetal bovine serum, glutamine, and antibiotics, at 37 °C and 5% CO₂. PBMCs were treated with DUL (100 µg/mL) for 24 h. Cisplatin (20 mM) was used as a positive control for cytotoxicity. Viability was determined by staining with 3-(4,5-dimethylthiazol-2-yl)−2,5-diphenyltetrazolium bromide (MTT), adding 150 µL of MTT at 0.5 mg/mL for 3 h and 150 µL of DMSO for solubilization. Absorbance levels were measured at 595 nm and expressed as a percentage relative to the negative control [18]. The selectivity index (SI) was calculated as the ratio of the IC₅₀ value to the MIC for each isolate [19].

### Tests performed to evaluate the possible mechanism of action

Four representative strains of *Candida* spp. were used. The isolates were incubated in yeast nitrogen dextrose (YND) medium (Becton Dickinson, CA, USA) for 24 h at 37 °C. The cells were then washed three times with 0.85% saline solution and resuspended in RPMI 1640 buffered with MOPS (Sigma-Aldrich, MO, USA), attaining a final inoculum of 10⁶ cells/mL. DUL was evaluated at MIC and 2× MIC concentrations of each strain. FLC was evaluated at MIC, AMB at a concentration of 4 µg/mL, and the DUL + AMB combination was evaluated at MIC. AMB was used as a positive control for cell death, whereas untreated cells were used as the negative control [20, 21].

### Cell viability assessment

This was assessed by propidium iodide (PI), 2 mg/L for 24 h after exposure to DUL using a FACSCalibur flow cytometer (Becton Dickinson, San Jose, CA, USA). Ten thousand events were analyzed per experiment, with cellular debris excluded from the analysis [20, 22].

### Investigation of phosphatidylserine externalization

Cells were incubated with DUL and centrifuged, then digested with 2 mg/L of zymolyase (Seikagaku Corp., Abingdon, UK) in potassium phosphate buffer (1 M sorbitol, pH 6.0) for 2 h at 30 °C. Protoplasts were stained with FITC- and PI-labeled annexin V using an apoptosis detection kit (Nexin Kit, Guava Technologies) and a FACSCalibur flow cytometer (Becton Dickinson, San Jose, CA, USA). 10,000 events were analyzed per experiment, with cellular debris excluded from the analysis [20, 23].

### Evaluation of transmembrane depolarization potential (Δψm)

Cells were washed with phosphate-buffered saline (PBS) and incubated with rhodamine 123 (Rho) at 5 mg/L at 37 °C for 30 min in the absence of light, using a FACSCalibur flow cytometer (Becton Dickinson, San Jose, CA, USA). Rho retention determined the ΔΨm, and 10,000 events were analyzed, with exclusion of cellular debris [20, 22, 23].

### Alkaline comet assay

Cells were centrifuged, washed and resuspended in a buffer solution (sorbitol and KH₂PO₄). Cells were then mixed with agarose, spread on slides, and incubated for 20 min at 30 °C, thus disintegrating the cell walls and forming spheroplasts. In a cold room (8 to 10 °C), the slides were incubated for 1 h in a solution containing NaOH, NaCl, lauryl, sarcosine, and EDTA, subjected to three washes of 20 min each, and then to electrophoresis, performed at 0.5 V/cm for 20 min with a current of 24 mA. The gels were neutralized using a Tris/HCl solution and incubated in ethyl alcohol at concentrations of 76% and 96% for 10 min each. The slides were dried, stained with ethidium bromide (1 mg/mL), and analyzed under a fluorescence microscope [20].

### ROS detection

Samples of treated *Candida* spp. cells were incubated with 20 µM of CM-H₂DCFDA [5-(and-6)-chloromethyl-2′,7′-dichlorodihydrofluorescein diacetate acetyl ester] (Sigma-Aldrich, MO, USA) for 30 min in the dark at 37 °C, then washed with PBS and analyzed for fluorescence using a FACSCalibur flow cytometer (Becton Dickinson, San Jose, CA, USA) [24–27].

### Determination of reduced glutathione (GSH), oxidized glutathione (GSSG), and the GSH/GSSG ratio

The assay evaluated the glutathione antioxidant system in *Candida* spp. strains. Total glutathione levels (GSH + GSSG) were determined by a spectrophotometric method based on the DTNB reaction, which generates 5-thio-2-nitrobenzoate (TNB). After 24 h of treatment, the cells were washed with cold PBS and lysed by sonication in a buffer containing sodium phosphate (0.1 M) and EDTA (5 mM), pH 8.0. The cell extract was precipitated with 2 M HClO₄ containing EDTA (4 mM) and centrifuged at 8000 g for 15 min at 4 °C. The supernatant was neutralized with 2 M KOH and subjected to further centrifugation. Quantification was performed by adding NADPH (4 mg/mL), glutathione reductase (6 U/mL), and DTNB (1.5 mg/mL), prepared in phosphate-EDTA buffer, to the supernatant. TNB formation was monitored at 412 nm and the results are expressed as µg of glutathione per mg of protein. For GSSG determination, samples were pretreated with 4-vinylpyridine (0.1% v/v) for 1 h at room temperature, allowing GSH derivatization. The GSH concentration was obtained by the difference between total glutathione and GSSG, and the GSH/GSSG ratio was subsequently calculated [27, 28].

### Evaluation of the survival of mutant strains of *C. albicans* in the presence of DUL

Cap1 and Gpx3 play important roles in the response to oxidative stress. Cap1 is a transcription factor involved in the oxidative stress response, whereas Gpx3 is a glutathione peroxidase responsible for peroxide sensing and detoxification. Mutant strains of *C. albicans* deficient in Cap1 (*cap1Δ*) and Gpx3 (*gpx3Δ*) were included in addition to the wild-type strain (WT). They were cultured at 30 °C in YPD medium (1% yeast extract, 2% bacterial peptone, 2% glucose). To evaluate the survival of *C. albicans*, cells in the exponential phase were diluted in YPD (10⁴ cells), treated with the compounds under study, and plated on YPD agar for quantification of colony-forming units (CFU). DUL was evaluated from 5 to 60 µg/mL for 24 h. For WT, H₂O₂ (positive control) was used at 0.5 mM for 30 min. The plates were incubated at 30 °C for 24 h and survival (%) is expressed relative to untreated control cultures [20, 29, 30]. (Table 1).

**Table 1:** Mutant strains used in the study

Strain	Relevant genotype	Source
WT (JC747)	SN148 + CIp30 (*URA3*,* HIS1*,* ARG4*)	da Silva Dantas et al., [31]
*cap1Δ* (JC842)	*cap1::loxP-HIS1-loxP/cap1::loxP*-*ARG4-loxP*, CIp20 (*HIS1*,* URA3*)	da Silva Dantas et al., [31]
*gpx3Δ* (JC1317)	*gpx3::loxP*-HIS1-*loxP/gpx3::loxP*-ARG4-*loxP* CIp20 (*HIS1*,* URA3*)	Patterson et al., [32]

Source: da Silva Dantas et al. [31]; Patterson et al. [32]

### Determination of activity against mature and forming *Candida* spp. biofilms

Four *Candida* spp. strains were selected and biofilms were formed according to Pierce et al. [33]. They were seeded on Sabouraud dextrose agar, incubated for 24 h at 35 ± 2 °C, suspended in 5 mL of YND and re-incubated under the same conditions. They were centrifuged at 3,000 g for 5 min and washed three times with PBS. The cell density was 0.5 on the McFarland scale in RPMI medium. For the developing biofilms, the inoculum and DUL were incubated simultaneously for 48 h. For the mature biofilms, the inocula were incubated for the same period before the DUL was added, followed by 24 h of incubation. To check cell viability, MTT (1 mg/mL) (Sigma, USA) was used. The supernatant was removed and DMSO was added. The reading was performed with a Smartreader™ 96 microplate reader (Accuris Instruments, NJ, USA) at 570 nm. The reduction in growth was compared to the well corresponding to 100% growth, without the presence of drugs [21].

### Data analysis

The assays were performed in triplicate. MIC and FICI values were obtained by arithmetic means. The action mechanism data were subjected to two-way analysis of variance (ANOVA) followed by Dunnett’s test (*p* < 0.05). For biofilms, ANOVA followed by Tukey’s test (*p* < 0.05) was used. The GraphPad Prism program (version 8 for Windows, GraphPad Software, La Jolla, CA, USA) was used for all statistical analyses.

## Results

### DUL had antifungal activity against *Candida* spp

DUL had MICs between 16 and 128 µg/mL. Among the 14 clinical strains, 50% were resistant to FLC, and of these 85.71% showed MICs for DUL ≤ 64 µg/mL. For FLC, MICs ranged from 0.3 to 64 µg/mL, for ITC from 0.1 to 2 µg/mL, and for AMB from 0.5 to 2 µg/mL. DUL exhibited fungicidal effects against all strains, with MFC values ranging from 26.6 to 213.3 µg/mL and the MFC/MIC ratio showing tolerance levels below 4 (Table 2).

**Table 2:** Antifungal action of DUL and tolerance level analysis

Strainsᵃ	MIC 50%ᵇ (µg/mL)				MFC^c^ (µg/mL)	MIC 100%^b^ (µg/mL)	MFC/MIC	Interpretation
	DUL	FLC	AMB	ITC				
*C. parapsilosis* ATCC 22,019	106.6	2.0	1.0	0.5	170.6	128	1.3	Fungicide
*C. krusei* ATCC 6258	21.3	32.0	1.0	0.5	32	32	1	Fungicide
*C. auris* 01256P*	26.6	1.6	1.0	0.3	32	32	1	Fungicide
*C. albicans* 1	128.0	21.3	0.5	1.0	128	128	1	Fungicide
*C. parapsilosis* 2*	32.0	26.6	1.0	0.1	32	42.6	0.75	Fungicide
*C. tropicalis* 1	128.0	0.5	1.0	0.3	170.6	128	1.3	Fungicide
*C. parapsilosis* 3	32.0	1.0	0.5	0.3	42.6	53.3	0.79	Fungicide
*C. albicans* 2	106.6	0.4	1.0	0.3	213.3	128	1.6	Fungicide
*C. albicans* 3*	16.0	32.0	2.0	1.0	32	32	1	Fungicide
*C. parapsilosis* 4	32.0	32.0	2.0	2.0	106.6	64	1.6	Fungicide
*C. glabrata* 1	16.0	2.0	2.0	0.5	26.6	16	1.6	Fungicide
*C. albicans* 4	128.0	0.3	0.5	0.3	213.3	128	1.6	Fungicide
*C. tropicalis* 2*	16.0	32.0	2.0	1.0	32	32	1	Fungicide
*C. glabrata* 2	42.6	64.0	2.0	2.0	64	64	1	Fungicide

DUL: duloxetine; FLC: fluconazole; AMB: amphotericin; ITC: itraconazole

^a^*Candida* spp. strains belonging to the mycotheca of the Laboratory of Bioprospecting of Antimicrobial Molecules (LABIMAN)

^b^Minimum Inhibitory Concentration individually

^c^Minimum Fungicidal Concentration of DUL

*Strains used for mechanism of action tests and biofilm assays

### The association of DUL with antifungals resulted in different types of drug interactions

For the combination of DUL with FLC, 92.8% of the strains were classified as having indifferent interactions and 7.2% as antagonistic. The combination of DUL with ITC resulted in 92.8% indifferent interactions and 7.2% additive interactions. The combination of DUL with AMB produced 92.8% synergistic and 7.2% additive strains (Table 3).

**Table 3:** Antifungal activity of DUL combined with conventional antifungals and their interactions

Strainsᵃ	MIC 50%^b^ (µg/mL)									
	DUL/FLC	FICI^c^	Interpretation	DUL/AMB	FICI^c^	Interpretation	DUL/ITC	FICI^c^		Interpretation
*C. parapsilosis* ATCC 22,019	106.6/2.0	2.00	Indifferent	6.7/0.1	0.13	Synergistic	53.3/0.3	1.00	Indifferent	
*C. krusei* ATCC 6258	85.2/16.0	4.50	Antagonistic	8.9/0.4	0.83	Additive	32.0/0.8	3.00	Indifferent	
*C. auris* 01256P	17.7/2.7	2.33	Indifferent	4.4/0.2	0.33	Synergistic	13.3/0.1	1.00	Indifferent	
*C. albicans* 1	85.3/21.3	1.67	Indifferent	21.3/0.1	0.33	Synergistic	64.0/0.5	1.00	Indifferent	
*C. parapsilosis 2*	64.0/32.0	3.20	Indifferent	3.3/0.2	0.31	Synergistic	24.0/0.2	2.25	Indifferent	
*C. tropicalis* 1	85.3/0.2	1.00	Indifferent	13.3/0.1	0.21	Synergistic	64.0/0.1	1.00	Indifferent	
*C. parapsilosis* 3	32.0/1.0	2.00	Indifferent	4.0/0	0.19	Synergistic	32.0/0.3	2.00	Indifferent	
*C. albicans* 2	88.8/0.4	1.88	Indifferent	5.6/0.1	0.10	Synergistic	53.3/0.1	1.00	Indifferent	
*C. albicans* 3	21.3/16.0	1.83	Indifferent	1.0/0.1	0.13	Synergistic	16.0/1.0	2.00	Indifferent	
*C. parapsilosis* 4	32.0/32.0	2.00	Indifferent	2.3/0.1	0.15	Synergistic	32.0/2.0	2.00	Indifferent	
*C. glabrata* 1	16.0/2.0	2.00	Indifferent	4.0/0.5	0.50	Synergistic	16.0/0.5	2.00	Indifferent	
*C. albicans* 4	85.3/0.3	2.00	Indifferent	21.3/0.1	0.33	Synergistic	48.0/0.1	0.75	Aditivo	
*C. tropicalis* 2	16.0/320	2.00	Indifferent	1.0/0.1	0.13	Synergistic	16.0/1.0	2.00	Indifferent	
*C. glabrata* 2	21.3/32.0	1.00	Indifferent	7.1/0.3	0.33	Synergistic	42.6/2.0	2.00	Indifferent	

DUL: duloxetine; FLC: fluconazole; AMB: amphotericin; ITC: itraconazole

^a^*Candida* spp. strains belonging to the mycotheca of the Laboratory of Bioprospecting of Antimicrobial Molecules (LABIMAN)

^b^Associated Minimum Inhibitory Concentration

^c^Fractional Inhibitory Concentration Index (FICI)

#### DUL demonstrated safety in PBMCs

**Fig. 1:**
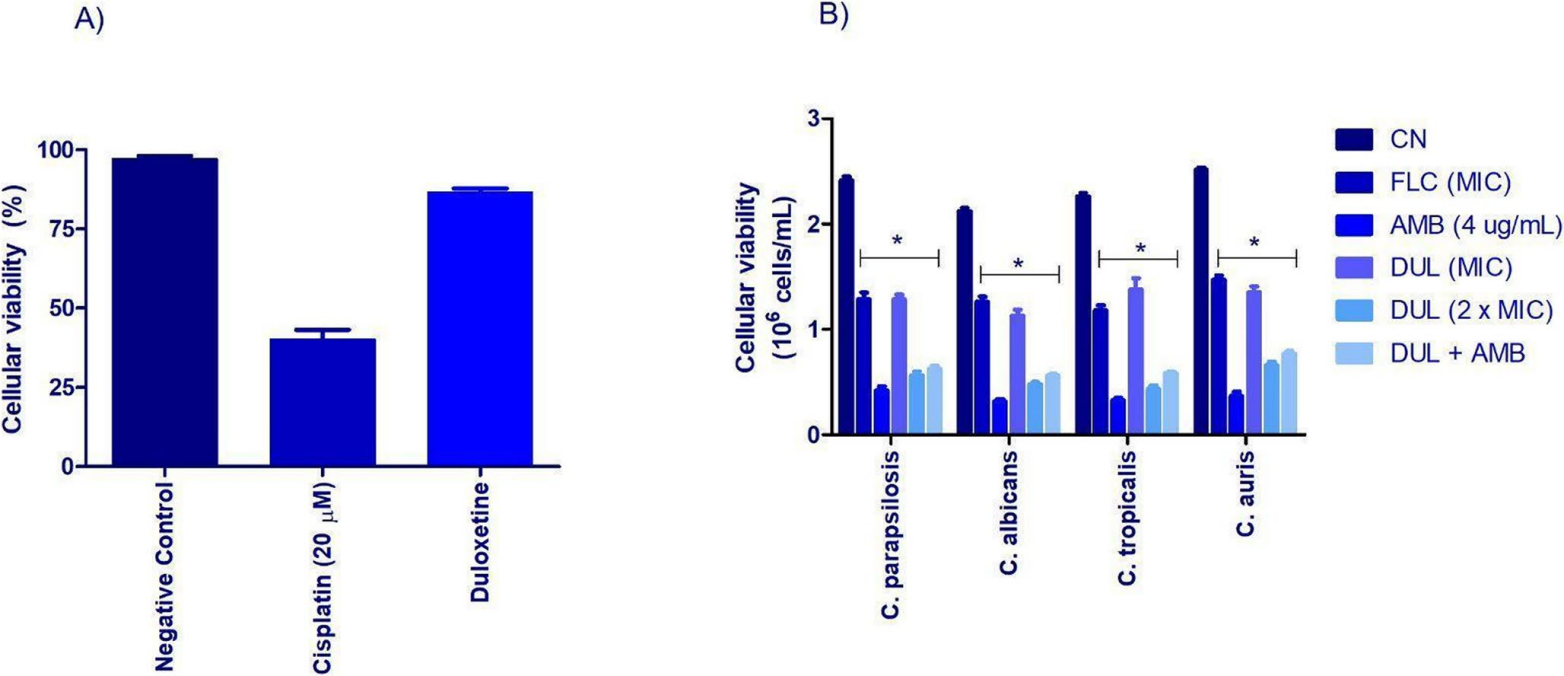
(**A**) Evaluation of DUL cytotoxicity in PBMCs. (**B**) Evaluation of DUL cell viability against *Candida* spp. with * *p* < 0.05 indicating significance compared to the control. Untreated cells were used as the negative control, whereas AMB (4 µg/mL) was used as the positive death control. Data are presented as the mean ± SD of at least three independent biological replicates, each performed in technical replicates

DUL exhibited an IC₅₀ >100 µg/mL in PBMCs, with an estimated SI ranging from > 0.78 to > 6.25 (Fig. 1A).

#### DUL reduced the numerical density of viable cells in *Candida* spp.

The effect was significant (*p* < 0.05) compared to the negative control of all concentrations tested. The DUL + AMB combination significantly reduced cell viability in all *Candida* species evaluated (Fig. 1B) (Online Resources 3 and 4).

#### DUL promoted phosphatidylserine externalization in Candida spp.

**Fig. 2:**
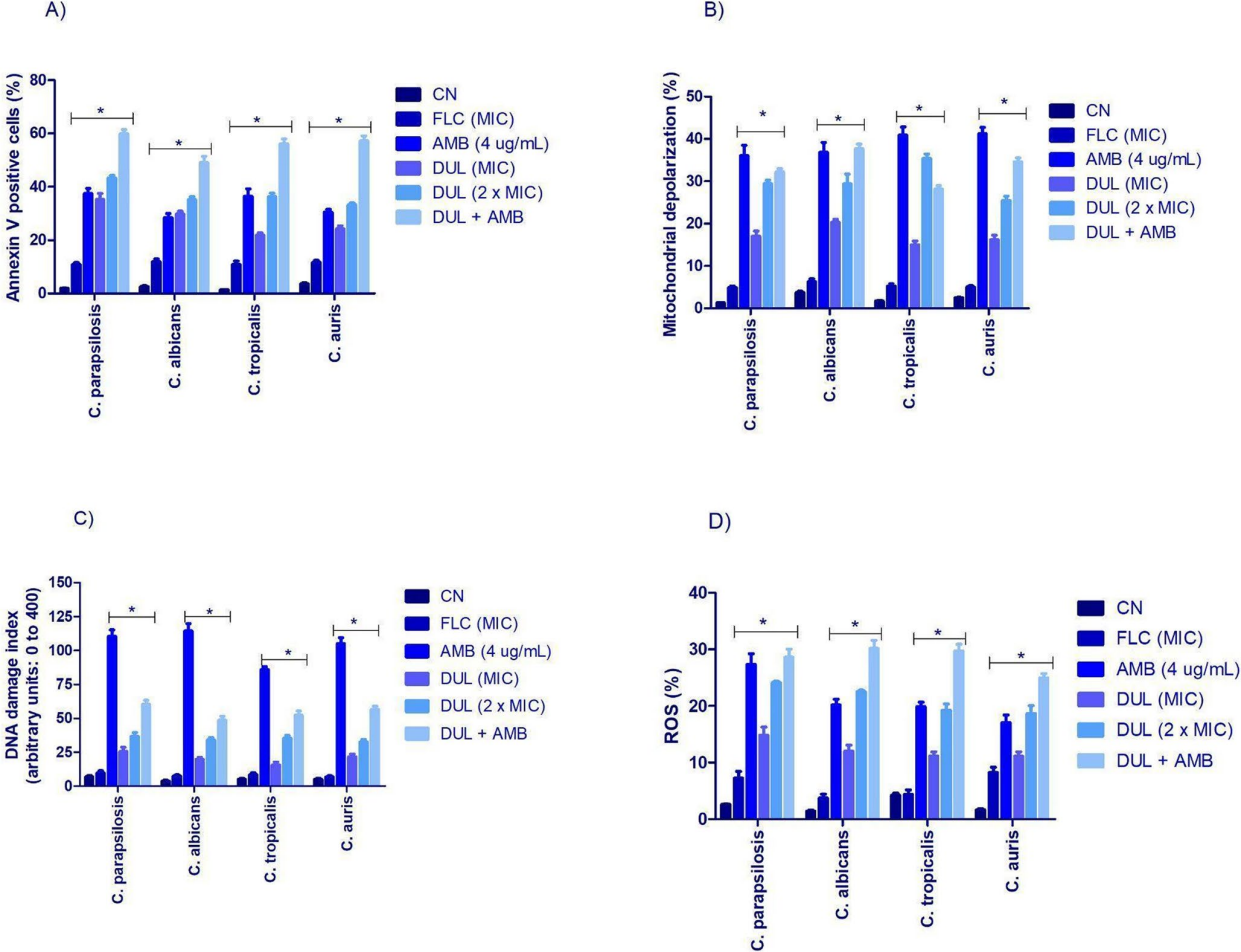
(**A**) Evaluation of phosphatidylserine externalization; (**B**) mitochondrial depolarization; (**C**) DNA damage; (**D**) reactive oxygen species. Cells were treated with DUL (MIC and 2x MIC), DUL + AMB (MIC), AMB (death control) (MIC = 4 µg/mL). Untreated cells (negative control) are compared with * (*p* < 0.05) indicating significance relative to the corresponding control. Data are presented as the mean ± SD of at least three independent biological replicates, each performed in technical replicates

Cells labeled with Annexin V had a higher percentage than the negative control (*p* < 0.05), mainly in the association of AMB and DUL. For FLC, the percentages were much lower, but still significant (Fig. 2A) (Online Resources 1 and 2).

#### DUL generated mitochondrial depolarization in *Candida* spp.

All strains caused significant mitochondrial depolarization (*p* < 0.05) when compared to the control. In general, AMB and the association with DUL showed the highest levels of depolarization. FLC did not have a significant effect on *Candida* spp. strains (Fig. 2B) (Online Resources 1 and 2).

#### DUL caused DNA damage in *Candida* spp.

DNA damage was observed in all *Candida* species when compared to the control. AMB had the highest values. DUL exhibited a dose-dependent effect, with greater damage at 2× MIC. The DUL + AMB combination resulted in an additional increase in DNA damage compared to DUL alone. Statistically significant differences (*p* < 0.05) were observed between the groups and their respective controls (Fig. 2C) (Online Resources 1 and 2).

#### DUL induced ROS production in *Candida* spp.

Treatment with DUL caused high levels of ROS (*p* < 0.05) in comparison with the negative control in all strains. For the association of AMB and DUL, these levels were even higher. For FLC, the levels were much lower and in 50% of the strains tested the values were not significant (Fig. 2D) (Online Resources 3 and 4).

#### DUL induced GSH reduction in *Candida* species

**Fig. 3:**
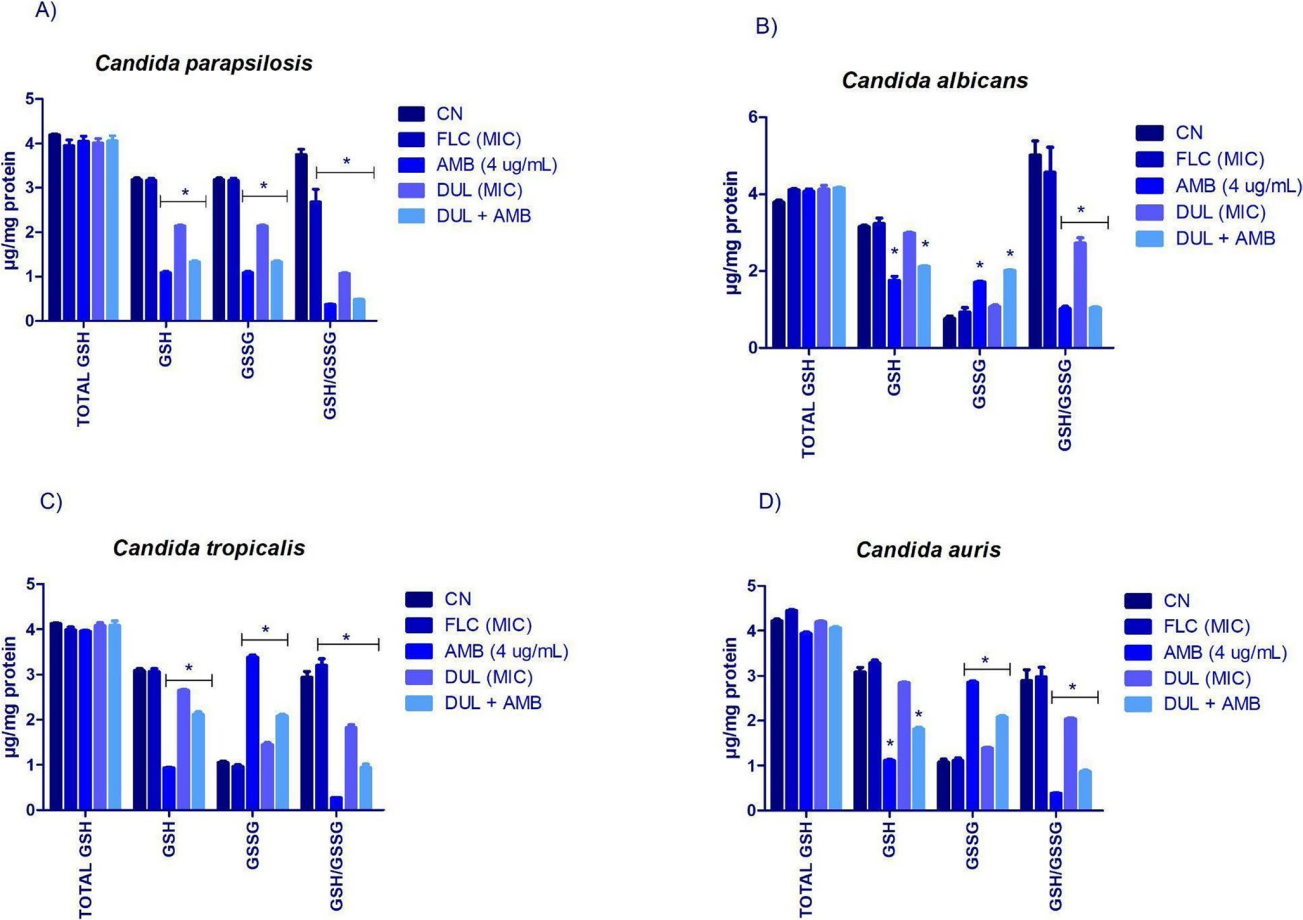
Effect of DUL alone and associated with AMB against strains of *C. parapsilosis* (**A**), *C. albicans* (**B**), *C. tropicalis* (**C**) and *C. auris* (**D**) on GSH, GSSG and the GSH/GSSG ratio. AMB (4 µg/mL) was used as the positive death control, whereas untreated cells (negative control) are compared with * (*p* < 0.05) indicating significance in relation to the corresponding control. Data are presented as the mean ± SD of at least three independent biological replicates, each performed in technical replicates

The control and FLC-treated groups presented higher GSH levels and higher GSH/GSSG ratios, indicating less oxidative stress. Treatment with DUL resulted in reduced GSH levels and increased GSSG in almost all species analyzed, evidencing the induction of oxidative stress. AMB also promoted changes, but to a lesser extent compared to DUL. The combination of DUL with AMB intensified these effects, promoting a more pronounced reduction in GSH levels, an increase in GSSG, and a decrease in the GSH/GSSG ratio. These findings suggest a potentiating effect in the induction of oxidative stress (Fig. 3) (Online Resource 5).

#### DUL reduced the survival rate of mutant strains of *C. albicans*

**Fig. 4:**
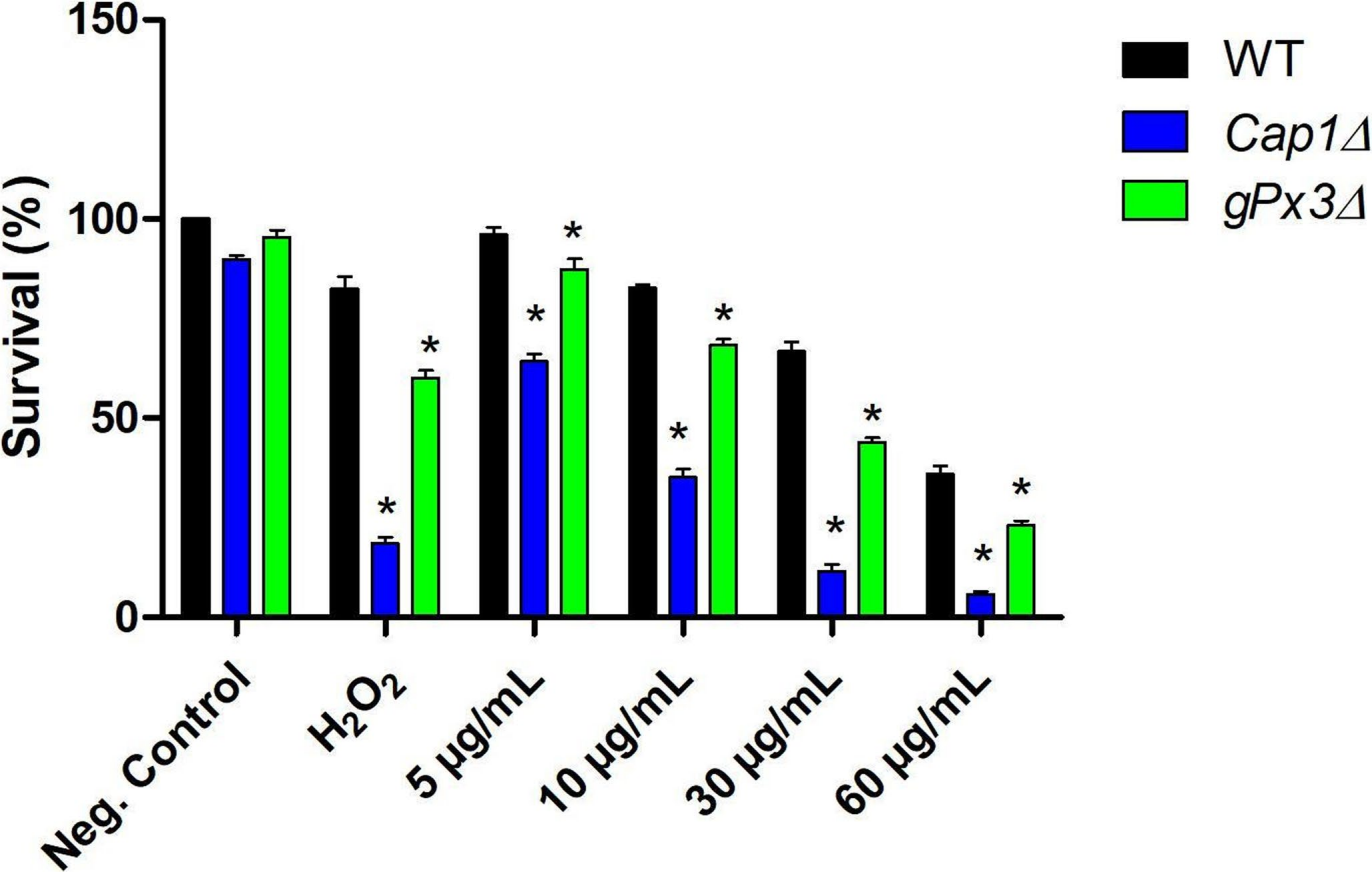
Evaluation of the survival of mutant strains of *C. albicans*. Mutant cells were treated with H₂O₂ (5 mM) and with increasing concentrations of DUL; the WT strain was used as a control; *cap1Δ*: strain with deletion of the CAP1 gene; *gpx3Δ*: strain with deletion of the GPX3 gene; * (*p* < 0.05) indicates significance in relation to the corresponding control. Data are presented as the mean ± SD of at least three independent biological replicates, each performed in technical replicates

All told, 82.4% of the WT strain cells survived, while the mutants showed greater sensitivity to oxidative stress, with survival rates of 60.1% for gpx3Δ and 18.5% for cap1Δ. For the lowest concentration of DUL tested (5 µg/mL), a high survival rate was observed for all strains, with 96.01% for WT, 64.1% for cap1Δ and 87.2% for gpx3Δ. With the increase in concentration to 10 µg/mL, these survival rates decreased to 82.7% in WT, 35.2% in cap1Δ and 68.3% in gpx3Δ. At 30 µg/mL, survival declined to 66.6% in WT, 11.5% in cap1Δ, and 43.8% in gpx3Δ. At the highest concentration tested (60 µg/mL), the lowest survival rates among the strains were observed, with 35.9% for WT, 5.6% for cap1Δ, and 23% for gpx3Δ (Fig. 4) (Online Resource 6).

#### DUL acts against mature and developing biofilms of *Candida* spp.

**Fig. 5:**
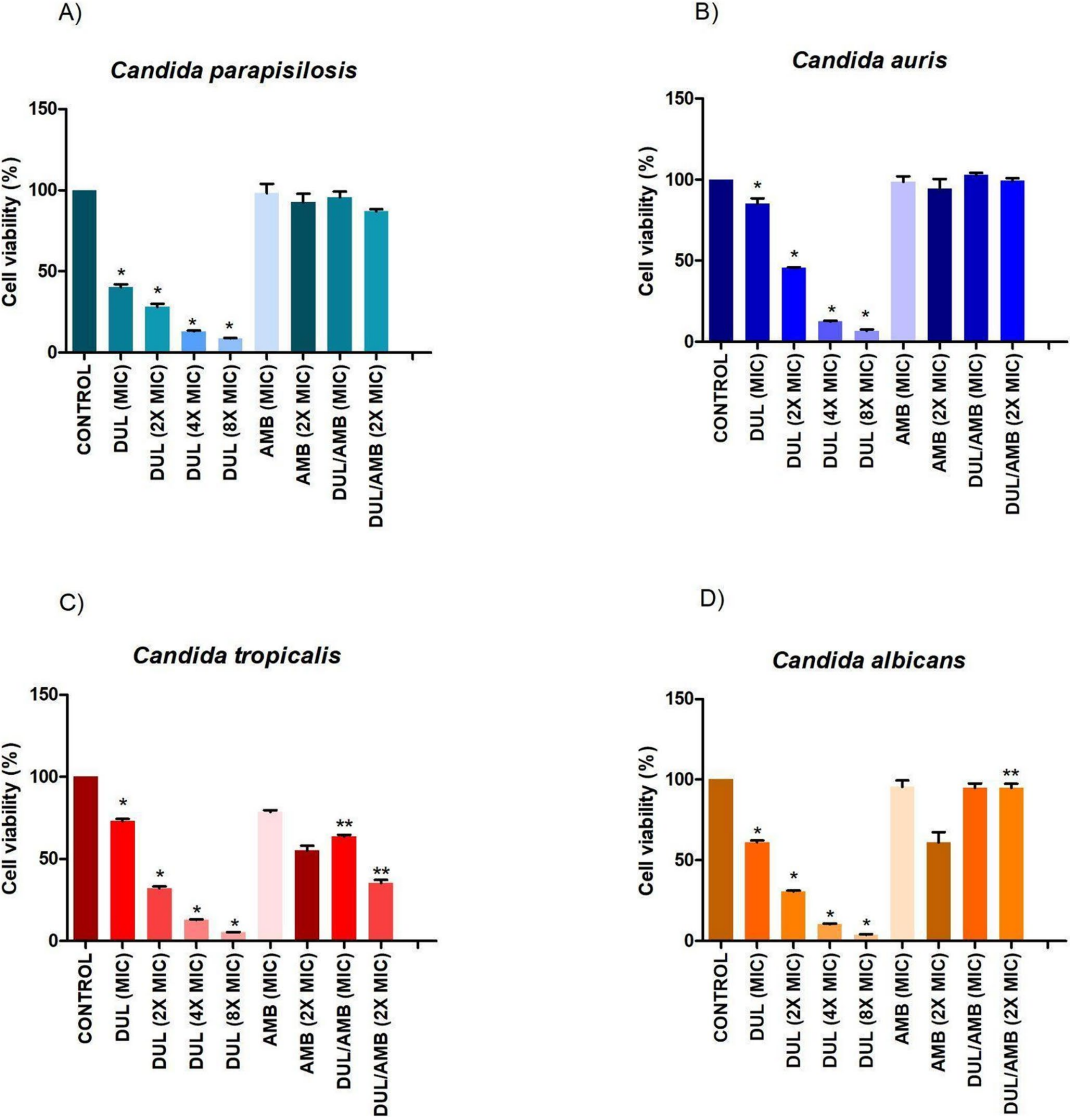
Evaluation of DUL alone and associated with AMB in mature biofilms against *C. parapsilosis* (**A**), *C. auris* (**B**), *C. tropicalis* (**C**) and *C. albicans* (**D**). The concentrations tested were: DUL alone at concentrations of MIC, 2× MIC, 4× MIC, and 8× MIC; AMB at concentrations of MIC and 2× MIC; and the DUL + AMB combination at concentrations of MIC and 2× MIC, with * (*p* < 0.05) indicating significance in relation to the corresponding control and with ** (*p* < 0.05) indicating significance in relation to its respective isolated concentration. Untreated biofilms served as the negative control, and AMB at its MIC was used as the reference antifungal treatment. Data are presented as the mean ± SD of at least three independent biological replicates, each performed in technical replicates

**Fig. 6:**
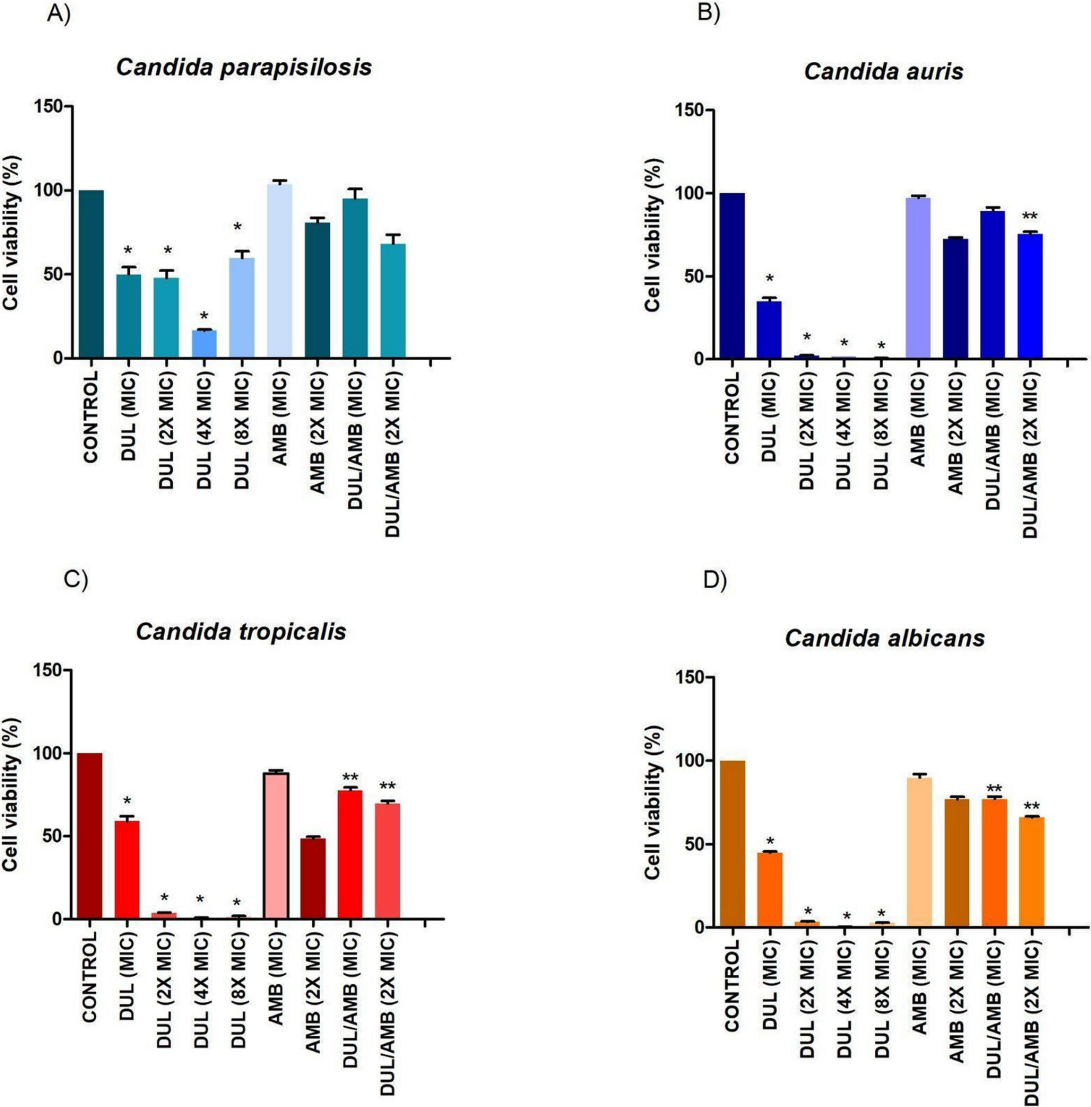
Evaluation of isolated DUL and associated with AMB in biofilm formation against *C. parapsilosis* (**A**), *C. auris* (**B**), *C. tropicalis* (**C**) and *C. albicans* (**D**). The concentrations tested were: DUL alone at concentrations of MIC, 2× MIC, 4× MIC, and 8× MIC; AMB at concentrations of MIC and 2× MIC; and the DUL + AMB combination at concentrations of MIC and 2× MIC, with * (*p* < 0.05) indicating significance in relation to the corresponding control and with ** (*p* < 0.05) indicating significance in relation to its respective isolated concentration. Untreated biofilms served as the negative control, and AMB at its MIC was used as the reference antifungal treatment. Data are presented as the mean ± SD of at least three independent biological replicates, each performed in technical replicates

DUL significantly reduced (*p* < 0.05) the viability of mature and forming biofilms when compared to the control. In mature biofilms, its association with AMB did not generate a significant reduction in most strains, occurring only in *C. tropicalis* and *C. albicans* at MIC x2 (Fig. 5). In the association with AMB in developing biofilms, there was a significant reduction in most strains (Fig. 6) (Online Resources 7 and 8).

## Discussion

Fungal infections are an important public health problem, due to their increase in recent decades and high morbidity and mortality rates, reinforcing the need to seek alternative treatment strategies [34]. The anti-*Candida* potential of DUL was evaluated, which demonstrated an antifungal effect in FLC-sensitive and resistant isolates of different species of this genus, also exhibiting a fungicidal profile.

Momeni et al. [35] reported that the antifungal effect of DUL was verified from 40 µg/mL against FLC-resistant *C. albicans* strains, with synergy in the DUL + FLC combination. In the present study, we observed lower concentrations and a predominance of indifferent interactions of FLC. These discrepancies may reflect differences in the experimental design, including DUL preparation, the strains evaluated, and the intrinsic susceptibility of the isolates.

Menezes et al. [36] observed an antifungal effect of DUL against Cryptococcus neoformans strains. They reported MIC and MFC of 18.5 µg/mL, a finding that that is corroborated by our results involving the MFC, suggesting fungicidal behavior. Regarding the association between DUL and the antifungals ITC and AMB, there are no data available in the literature. It is noteworthy that in the DUL + AMB associations, there was predominant synergism, a relevant aspect considering the toxicity associated with AMB.

Regarding the possible mechanism of action, a significant reduction in the cell viability of *Candida* spp. associated with oxidative stress was demonstrated, indicated by the modulation of the GSH/GSSG ratio. The DUL + AMB combination intensified this redox imbalance, suggesting a possible synergistic effect. These results indicate that increased ROS can cause changes in the oxidative state, affecting cell signaling cascades [37].

Treatment with DUL also generated increased ROS accompanied by significant mitochondrial depolarization, with the DUL + AMB association showing high levels, which reinforces the potentiation of this combination. Furthermore, an increase in Annexin V staining was observed with the treatments, indicating cell death. Therefore, isolated DUL induced apoptosis-like events consistent with programmed cell death in all strains tested, and its combination with AMB enhanced this effect. These results are consistent with apoptosis-like processes in fungi, characterized by oxidative damage, mitochondrial dysfunction, and phosphatidylserine externalization [38].

Another relevant factor in oxidative stress in *Candida* spp., especially in *C. albicans*, is the transcription factor Cap1Δ, an important regulator of the oxidative response [39]. Strains deficient in ROS sensors (cap1Δ) or detoxifiers (gpx3Δ), with GPX3 being determinant of resistance to oxidative stress [40], promoted marked decreases in survival as the concentration increased, mainly in cap1Δ, suggesting a more determinant role of Cap1 in DUL tolerance.

Another aspect also observed was that DUL interacts with DNA, sensitizing this molecule to structural damage under stress conditions, which is consistent with our results in the present study [41]. In general, all treatments promoted a significant increase in DNA damage, with the effect being dose-dependent for DUL. These findings on the possible antifungal mechanism of action corroborate those reported by Farias Cabral et al. [42], in which an increase in ROS associated with the induction of DNA lesions was also observed.

In addition to planktonic cells, biofilms are also important since they are one of the main virulence factors in the development of fungal infections [43]. Rehem et al. [44] investigated the antibiofilm activity of DUL against *Cryptococcus* spp. strains, in which they obtained a reduction in biomass at a level comparable to that of AMB, with a significant decrease (85%) for *C. neoformans* biofilms. In this sense, the authors also verified that the metabolic activity of *C. neoformans* and *C. gattii* biofilms was greatly reduced (99%) after treatment with DUL.

In this present study, mature and developing *Candida* spp. biofilms showed a significant reduction in treatment with DUL. In association with AMB, the mature biofilm did not show significance in most isolates, unlike the developing biofilm, for which there were more significant data, such as in *C. tropicalis* and *C. albican*s, observed in DUL/AMB MIC and DUL/AMB 2×MIC, for *C. auris* only in DUL/AMB 2×MIC. This result can be explained by the complexity of the structure of the already formed biofilms, which have greater resistance to treatments, unlike the developing biofilms, in which the structure is still being formed and facilitates the action of the association [43].

The results obtained for cell viability in PBMCs demonstrated safety in human cells. Furthermore, transdermal formulations of DUL have already shown sustained release and therapeutic potential [45], while coatings containing DUL on catheter surfaces have demonstrated anti-adhesive activity and reduction of bacterial biofilm formation [46]. Estimated SI values support DUL safety. These findings reinforce the potential for repositioning DUL for topical applications and in medical devices aimed at combating biofilms.

Therefore, it is important to reiterate the potential of DUL as a promising candidate for antifungal repositioning, with potential for development for topical use in the treatment of skin infections, since the *Candida* genus is one of the main causes. In view of this, more studies should be carried out involving in vivo assays.

## Conclusion

DUL is a promising antifungal, exhibiting significant activity against FLC-resistant *Candida* spp. strains, in planktonic cells and biofilms. In addition, it demonstrated safety in human cells. Its mechanisms of action appear to be strongly associated with the induction of oxidative stress, mitochondrial depolarization, apoptosis-like events consistent with programmed cell death, and DNA damage. These findings reinforce the potential of DUL as a candidate for pharmacological repositioning for the treatment of fungal infections.

## Supplementary Information

Below is the link to the electronic supplementary material.

Supplementary Material 1 (DOCX 10.1 KB)

Supplementary Material 2 (DOCX 10.1 KB)

Supplementary Material 3 (DOCX 9.81 KB)

Supplementary Material 4 (DOCX 9.76 KB)

Supplementary Material 5 (DOCX 10.0 KB)

Supplementary Material 6 (DOCX 9.55 KB)

Supplementary Material 7 (DOCX 9.76 KB)

Supplementary Material 8 (DOCX 9.73 KB)

## Data Availability

All data generated or analyzed during this study are included in this article [and in its supplementary information files].
