# Supplementary material for: Duloxetine repositioning: Investigation of antifungal and antibiofilm activity against fluconazole-sensitive and resistant Candida Spp. strains
Source: Braz J Microbiol. 2026 Aug 3;57(1):226. doi: 10.1007/s42770-026-02038-z (PMC13433714; doi:10.1007/s42770-026-02038-z)
Supplement: Supplementary file 1 [file 42770_2026_2038_MOESM1_ESM.docx]

**Brazilian Journal of Microbiology**

Duloxetine Repositioning: Investigation of Antifungal and Antibiofilm Activity against Fluconazole-Sensitive and Resistant *Candida* Spp. Strains

Sarah Alves Barbosa^1,2^; Dávylla Rênnia Saldanha Pinheiro^1,2^; Leilson Carvalho de Oliveira^1,2^, Lara Elloyse de Almeida Moreira^1,2^, Vitória Pessoa Farias Cabral^1,2^, Daniel Sampaio Rodrigues^1,2^, Beatriz Oliveira de Souza^1,2^, Hélio Vitoriano Nobre Júnior^1,2^; Livia Gurgel Do Amaral Valente Sá^1,2^; Bruno Coêlho Cavalcanti^2,4^; Islay Lima Magalhães^2^; José Roberto de Oliveira Ferreira^5^; Manoel Odorico de Moraes Filho^2,4^; João Batista de Andrade Neto^1,2,3^; Cecília Rocha da Silva^1,2^.

^1^Department of Clinical and Toxicological Analyses, Faculty of Pharmacy, Laboratory of Bioprospecting of Antimicrobial Molecules (LABIMAN), Federal University of Ceará, Fortaleza, CE, Brazil

^2^Nucleus for Drug Research and Development (NPDM), Federal University of Ceará, Fortaleza, CE, Brazil

^3^Christus University (UNICHRISTUS), Fortaleza, CE, Brazil

⁴Department of Physiology and Pharmacology, Faculty of Medicine, Federal University of Ceará, Fortaleza, CE, Brazil

⁵School of Medical Sciences, State University of Health Sciences of Alagoas (UNCISAL), Maceió, AL, Brazil.

* Corresponding author:

Telephone number: # 55 85 3366 8274

Fax: # 55 85 3366 8257

E-mail: labimanufc1@gmail.com.br

Online Resource 1 – Effects of isolated DUL and DUL combined with AMB on phosphatidylserine externalization, mitochondrial depolarization, and DNA damage in *C. albicans* and *C. auris*

*p < 0.05 compared to control by ANOVA followed by Dunnett test. Data are presented as means ± S.D. for three independent experiments. FLC: fluconazol; AMB: anfotericina B; DUL: duloxetina.

| Drug treatments |  | | *Candida*  *albicans* | | | | *Candida*  *auris* | | |
| --- | --- | --- | --- | --- | --- | --- | --- | --- | --- |
|  | Annexin V positive cells (%) | Mitochondrial depolarization (%) | | DNA  damage  index |  | Annexin V positive cells (%) | | Mitochondrial depolarization (%) | DNA  damage  index |
| Negative Control | 2.57 ± 0.98 | 3.77 ± 0.81 | | 3.66 ± 2.00 |  | 3.64 ± 1.16 | | 2.56 ± 0.27 | 5.11 ± 2.02 |
| FLC (MIC) | 11.98 ± 2.90* | 6.33 ± 1.88 | | 7.44 ± 2.55* |  | 11.64 ± 2.45* | | 5.05 ± 0.85 | 6.77 ± 2.90* |
| AMB (4 μg/mL) | 28.45 ± 4.44* | 36.87 ± 6.99* | | 114.4± 16.06* |  | 30.47 ± 3.29* | | 41.21 ± 4.56* | 105 ± 13.15* |
| DUL (MIC) | 29.83± 3.08* | 20.31 ± 2.11* | | 19.77 ± 4.23* |  | 24.29 ± 3.25* | | 16.23 ±3.14* | 21.55± 6.32* |
| DUL (2xMIC) | 35.16 ± 3.43* | 29.35 ± 7.08* | | 33.88 ± 5.64* |  | 33.29 ±2.09* | | 25.47± 3.07* | 32.55 ± 5.63* |
| DUL + AMB | 49.11 ± 6.97* | 37.39 ± 3.28* | | 48.55 ± 8.61* |  | 57.25 ± 5.59* | | 34.61 ± 2.68* | 56.66 ± 6.83* |
