# Supplementary material for: Duloxetine repositioning: Investigation of antifungal and antibiofilm activity against fluconazole-sensitive and resistant Candida Spp. strains
Source: Braz J Microbiol. 2026 Aug 3;57(1):226. doi: 10.1007/s42770-026-02038-z (PMC13433714; doi:10.1007/s42770-026-02038-z)
Supplement: Supplementary file 2 [file 42770_2026_2038_MOESM2_ESM.docx]

**Brazilian Journal of Microbiology**

Duloxetine Repositioning: Investigation of Antifungal and Antibiofilm Activity against Fluconazole-Sensitive and Resistant *Candida* Spp. Strains

Sarah Alves Barbosa^1,2^; Dávylla Rênnia Saldanha Pinheiro^1,2^; Leilson Carvalho de Oliveira^1,2^, Lara Elloyse de Almeida Moreira^1,2^, Vitória Pessoa Farias Cabral^1,2^, Daniel Sampaio Rodrigues^1,2^, Beatriz Oliveira de Souza^1,2^, Hélio Vitoriano Nobre Júnior^1,2^; Livia Gurgel Do Amaral Valente Sá^1,2^; Bruno Coêlho Cavalcanti^2,4^; Islay Lima Magalhães^2^; José Roberto de Oliveira Ferreira^5^; Manoel Odorico de Moraes Filho^2,4^; João Batista de Andrade Neto^1,2,3^; Cecília Rocha da Silva^1,2^.

^1^Department of Clinical and Toxicological Analyses, Faculty of Pharmacy, Laboratory of Bioprospecting of Antimicrobial Molecules (LABIMAN), Federal University of Ceará, Fortaleza, CE, Brazil

^2^Nucleus for Drug Research and Development (NPDM), Federal University of Ceará, Fortaleza, CE, Brazil

^3^Christus University (UNICHRISTUS), Fortaleza, CE, Brazil

⁴Department of Physiology and Pharmacology, Faculty of Medicine, Federal University of Ceará, Fortaleza, CE, Brazil

⁵School of Medical Sciences, State University of Health Sciences of Alagoas (UNCISAL), Maceió, AL, Brazil.

* Corresponding author:

Telephone number: # 55 85 3366 8274

Fax: # 55 85 3366 8257

E-mail: labimanufc1@gmail.com.br

Online Resource 2 – Effects of isolated DUL and DUL combined with AMB on phosphatidylserine externalization, mitochondrial depolarization, and DNA damage in *C. parapsilosis* and *C. tropicalis*

*p < 0.05 compared to control by ANOVA followed by Dunnett test. Data are presented as means ± S.D. for three independent experiments. FLC: fluconazol; AMB: anfotericina B; DUL: duloxetina.

| Drug treatments |  | | *Candida*  *parapsilosis* | | | | *Candida*  *tropicalis* | | |
| --- | --- | --- | --- | --- | --- | --- | --- | --- | --- |
|  | Annexin V positive cells (%) | Mitochondrial depolarization (%) | | DNA  damage  index |  | Annexin V positive cells (%) | | Mitochondrial depolarization (%) | DNA  damage  index |
| Negative Control | 1.89 ± 0.49 | 1.36 ± 0.13 | | 10.00 ± 2.89 |  | 1.37 ± 0.19 | | 1.72 ± 0.53 | 5.00 ± 1.93 |
| FLC (MIC) | 10.80 ± 2.37* | 4.94 ± 1.07 | | 87.00 ± 4.81* |  | 10.96 ± 3.55* | | 5.30 ± 1.38 | 82.67 ± 4.21* |
| AMB (4 μg/mL) | 37.51 ± 5.76* | 36.05 ± 7.46* | | 65.33 ± 14.62* |  | 36.44 ±8.43* | | 40.91 ± 5.66* | 55.33 ± 6.64* |
| DUL (MIC) | 35.23 ± 6.75* | 17.06 ± 3.67* | | 42.33 ± 9.55* |  | 21.94 ± 2.52* | | 15.00 ± 2.65* | 46.00 ± 6.39* |
| DUL (2xMIC) | 43.33 ± 2.91* | 29.46 ± 2.58* | | 35.33 ± 8.35* |  | 36.32 ± 3.83* | | 35.37 ± 3.14* | 42.67 ± 6.28* |
| DUL + AMB | 59.93 ± 4.63* | 32.21 ± 2.45* | | 5.33 ± 8.32* |  | 56.07 ± 5.68* | | 28.17 ± 2.40* | 13.67 ± 8.86* |
