# Supplementary material for: Duloxetine repositioning: Investigation of antifungal and antibiofilm activity against fluconazole-sensitive and resistant Candida Spp. strains
Source: Braz J Microbiol. 2026 Aug 3;57(1):226. doi: 10.1007/s42770-026-02038-z (PMC13433714; doi:10.1007/s42770-026-02038-z)
Supplement: Supplementary file 3 [file 42770_2026_2038_MOESM3_ESM.docx]

**Brazilian Journal of Microbiology**

Duloxetine Repositioning: Investigation of Antifungal and Antibiofilm Activity against Fluconazole-Sensitive and Resistant *Candida* Spp. Strains

Sarah Alves Barbosa^1,2^; Dávylla Rênnia Saldanha Pinheiro^1,2^; Leilson Carvalho de Oliveira^1,2^, Lara Elloyse de Almeida Moreira^1,2^, Vitória Pessoa Farias Cabral^1,2^, Daniel Sampaio Rodrigues^1,2^, Beatriz Oliveira de Souza^1,2^, Hélio Vitoriano Nobre Júnior^1,2^; Livia Gurgel Do Amaral Valente Sá^1,2^; Bruno Coêlho Cavalcanti^2,4^; Islay Lima Magalhães^2^; José Roberto de Oliveira Ferreira^5^; Manoel Odorico de Moraes Filho^2,4^; João Batista de Andrade Neto^1,2,3^; Cecília Rocha da Silva^1,2^.

^1^Department of Clinical and Toxicological Analyses, Faculty of Pharmacy, Laboratory of Bioprospecting of Antimicrobial Molecules (LABIMAN), Federal University of Ceará, Fortaleza, CE, Brazil

^2^Nucleus for Drug Research and Development (NPDM), Federal University of Ceará, Fortaleza, CE, Brazil

^3^Christus University (UNICHRISTUS), Fortaleza, CE, Brazil

⁴Department of Physiology and Pharmacology, Faculty of Medicine, Federal University of Ceará, Fortaleza, CE, Brazil

⁵School of Medical Sciences, State University of Health Sciences of Alagoas (UNCISAL), Maceió, AL, Brazil.

* Corresponding author:

Telephone number: # 55 85 3366 8274

Fax: # 55 85 3366 8257

E-mail: labimanufc1@gmail.com.br

Online Resource 3 – Effects of isolated DUL and DUL combined with AMB on cell viability and reactive oxygen species in *C. albicans* and *C. auris*

*p < 0.05 compared to control by ANOVA followed by Dunnett test. Data are presented as means ± S.D. for three independent experiments. FLC: fluconazol; AMB: anfotericina B; DUL: duloxetina.

| Drug treatments |  | | *Candida*  *albicans* | | | *Candida*  *auris* | |
| --- | --- | --- | --- | --- | --- | --- | --- |
|  | Cellular viability  (10⁶ cells/mL) | ROS (%) | |  | Cellular viability  (10⁶ cells/mL) | | ROS (%) |
| Negative Control | 2.12 ± 0.08 | 1.41 ± 0.49 | |  | 2.51 ± 0.07 | | 1.65 ± 0.50 |
| FLC (MIC) | 1.26 ± 0.15* | 3.73 ± 1.90 | |  | 1.46 ± 0.12* | | 8.24 ± 2.68* |
| AMB (4 μg/mL) | 0.31 ± 0.07* | 20.18 ± 3.15* | |  | 0.36 ± 0.13* | | 17.03 ± 4.09* |
| DUL (MIC) | 1.12 ± 0.16* | 12.03 ± 3.15* | |  | 1.35 ± 0.16* | | 11.13 ± 2.21* |
| DUL (2xMIC) | 0.47 ± 0.07* | 22.53 ± 0.68* | |  | 0.66 ± 0.10* | | 18.69 ± 4.03* |
| DUL + AMB | 0.56 ± 0.05* | 30.19 ± 4.31* | |  | 0.77 ± 0.06* | | 24.98 ± 2.18* |
