# Supplementary material for: Duloxetine repositioning: Investigation of antifungal and antibiofilm activity against fluconazole-sensitive and resistant Candida Spp. strains
Source: Braz J Microbiol. 2026 Aug 3;57(1):226. doi: 10.1007/s42770-026-02038-z (PMC13433714; doi:10.1007/s42770-026-02038-z)
Supplement: Supplementary file 4 [file 42770_2026_2038_MOESM4_ESM.docx]

**Brazilian Journal of Microbiology**

Duloxetine Repositioning: Investigation of Antifungal and Antibiofilm Activity Against Fluconazole-Sensitive and Resistant *Candida* Spp. Strains

Sarah Alves Barbosa^1,2^; Dávylla Rênnia Saldanha Pinheiro^1,2^; Leilson Carvalho de Oliveira^1,2^, Lara Elloyse de Almeida Moreira^1,2^, Vitória Pessoa Farias Cabral^1,2^, Daniel Sampaio Rodrigues^1,2^, Beatriz Oliveira de Souza^1,2^, Hélio Vitoriano Nobre Júnior^1,2^; Livia Gurgel Do Amaral Valente Sá^1,2^; Bruno Coêlho Cavalcanti^2,4^; Islay Lima Magalhães^2^; José Roberto de Oliveira Ferreira^5^; Manoel Odorico de Moraes Filho^2,4^; João Batista de Andrade Neto^1,2,3^; Cecília Rocha da Silva^1,2^.

^1^Department of Clinical and Toxicological Analyses, Faculty of Pharmacy, Laboratory of Bioprospecting of Antimicrobial Molecules (LABIMAN), Federal University of Ceará, Fortaleza, CE, Brazil

^2^Nucleus for Drug Research and Development (NPDM), Federal University of Ceará, Fortaleza, CE, Brazil

^3^Christus University (UNICHRISTUS), Fortaleza, CE, Brazil

⁴Department of Physiology and Pharmacology, Faculty of Medicine, Federal University of Ceará, Fortaleza, CE, Brazil

⁵School of Medical Sciences, State University of Health Sciences of Alagoas (UNCISAL), Maceió, AL, Brazil.

* Corresponding author:

Telephone number: # 55 85 3366 8274

Fax: # 55 85 3366 8257

E-mail: labimanufc1@gmail.com.br

Online Resource 4 – Effects of isolated DUL and DUL combined with AMB on cell viability and reactive oxygen species in *C. parapsilosis* and *C. tropicalis.*

*p < 0.05 compared to control by ANOVA followed by Dunnett test. Data are presented as means ± S.D. for three independent experiments. FLC: fluconazol; AMB: anfotericina B; DUL: duloxetina.

| Drug treatments |  | | *Candida*  *parapsilosis* | | | *Candida*  *tropicalis* | |
| --- | --- | --- | --- | --- | --- | --- | --- |
|  | Cellular viability  (10⁶ cells/mL) | ROS (%) | |  | Cellular viability  (10⁶ cells/mL) | | ROS (%) |
| Negative Control | 2.41 ± 0.10 | 3.77 ± 0.81 | |  | 2.26 ± 0.09 | | 2.56 ± 0.27 |
| FLC (MIC) | 1.28 ± 0.18* | 6.33 ± 1.88* | |  | 1.18 ± 0.14* | | 5.05 ± 0.85 |
| AMB (4 μg/mL) | 0.42 ± 0.11* | 36.87 ± 6.99* | |  | 0.32 ± 0.07* | | 41.21 ± 4.56* |
| DUL (MIC) | 1.28 ± 0.13* | 20.31 ± 2.11* | |  | 1.37 ± 0.32* | | 16.23 ±3.14* |
| DUL (2xMIC) | 0.56 ± 0.11* | 29.35 ± 7.08* | |  | 0.43 ± 0.08* | | 25.47± 3.07* |
| DUL + AMB | 0.62 ± 0.09* | 37.39 ± 3.28* | |  | 0.58 ± 0.04* | | 34.61 ± 2.68* |
