# Supplementary material for: Duloxetine repositioning: Investigation of antifungal and antibiofilm activity against fluconazole-sensitive and resistant Candida Spp. strains
Source: Braz J Microbiol. 2026 Aug 3;57(1):226. doi: 10.1007/s42770-026-02038-z (PMC13433714; doi:10.1007/s42770-026-02038-z)
Supplement: Supplementary file 5 [file 42770_2026_2038_MOESM5_ESM.docx]

**Brazilian Journal of Microbiology**

Duloxetine Repositioning: Investigation of Antifungal and Antibiofilm Activity against Fluconazole-Sensitive and Resistant *Candida* Spp. Strains

Sarah Alves Barbosa^1,2^; Dávylla Rênnia Saldanha Pinheiro^1,2^; Leilson Carvalho de Oliveira^1,2^, Lara Elloyse de Almeida Moreira^1,2^, Vitória Pessoa Farias Cabral^1,2^, Daniel Sampaio Rodrigues^1,2^, Beatriz Oliveira de Souza^1,2^, Hélio Vitoriano Nobre Júnior^1,2^; Livia Gurgel Do Amaral Valente Sá^1,2^; Bruno Coêlho Cavalcanti^2,4^; Islay Lima Magalhães^2^; José Roberto de Oliveira Ferreira^5^; Manoel Odorico de Moraes Filho^2,4^; João Batista de Andrade Neto^1,2,3^; Cecília Rocha da Silva^1,2^.

^1^Department of Clinical and Toxicological Analyses, Faculty of Pharmacy, Laboratory of Bioprospecting of Antimicrobial Molecules (LABIMAN), Federal University of Ceará, Fortaleza, CE, Brazil

^2^Nucleus for Drug Research and Development (NPDM), Federal University of Ceará, Fortaleza, CE, Brazil

^3^Christus University (UNICHRISTUS), Fortaleza, CE, Brazil

⁴Department of Physiology and Pharmacology, Faculty of Medicine, Federal University of Ceará, Fortaleza, CE, Brazil

⁵School of Medical Sciences, State University of Health Sciences of Alagoas (UNCISAL), Maceió, AL, Brazil.

* Corresponding author:

Telephone number: # 55 85 3366 8274

Fax: # 55 85 3366 8257

E-mail: labimanufc1@gmail.com.br

Online Resource 5 –Effects of DUL alone and in combination with AMB on GSH in *C. albicans* and *C. auris*

*p < 0.05 compared to control by ANOVA followed by Dunnett test. Data are presented as means ± S.D. for three independent experiments. FLC: fluconazol; AMB: anfotericina B; DUL: duloxetina.

| Drug treatments |  | | *Candida*  *albicans* | | | | | *Candida*  *auris* | | | |
| --- | --- | --- | --- | --- | --- | --- | --- | --- | --- | --- | --- |
|  | TOTAL GSH | GSH | | GSSG | GSH/GSSG |  | TOTAL GSH | | GSH | GSSG | GSH/GSSG |
| Neg. Control | 3.08 ± 0.07 | 3.15 ± 0.05 | | 0.76 ± 0.11 | 5.01 ± 0.65 |  | 4.22 ± 0.06 | | 3.08 ± 0.16 | 1.07 ± 0.10 | 2.89 ± 0.41 |
| FLC (MIC) | 4.11 ± 0.06 | 3.23 ± 0.25 | | 0.93 ± 0.20 | 4.57 ± 1.13 |  | 4.45 ± 0.03 | | 3.28 ± 0.10 | 1.11 ± 0.09 | 2.97 ± 0.37 |
| AMB (4 μg/mL) | 4.07 ± 0.11 | 1.76 ± 0,18* | | 1.71 ± 0.03* | 1.02 ± 0.09* |  | 3.94 ± 0.05 | | 1.11 ± 0.03* | 2.85 ± 0.05* | 0.38 ± 0.01* |
| DUL (MIC) | 4.13 ± 0.16 | 2.98 ± 0.04 | | 1.07 ± 0.06 | 2.72 ± 0.24* |  | 4.19 ± 0.03 | | 2.84 ± 0.02 | 1.39 ± 0.02* | 2.04 ± 0.03* |
| DUL + AMB | 4.15 ± 0.03 | 2.12 ± 0.03* | | 42.01 ± 0.02* | 1.04 ± 0.03* |  | 4.06 ± 0.04 | | 1.81 ± 0.06* | 2.07 ± 0.05* | 0.87 ± 0.04* |
