# Supplementary material for: Duloxetine repositioning: Investigation of antifungal and antibiofilm activity against fluconazole-sensitive and resistant Candida Spp. strains
Source: Braz J Microbiol. 2026 Aug 3;57(1):226. doi: 10.1007/s42770-026-02038-z (PMC13433714; doi:10.1007/s42770-026-02038-z)
Supplement: Supplementary file 6 [file 42770_2026_2038_MOESM6_ESM.docx]

**Brazilian Journal of Microbiology**

Duloxetine Repositioning: Investigation of Antifungal and Antibiofilm Activity against Fluconazole-Sensitive and Resistant *Candida* Spp. Strains

Sarah Alves Barbosa^1,2^; Dávylla Rênnia Saldanha Pinheiro^1,2^; Leilson Carvalho de Oliveira^1,2^, Lara Elloyse de Almeida Moreira^1,2^, Vitória Pessoa Farias Cabral^1,2^, Daniel Sampaio Rodrigues^1,2^, Beatriz Oliveira de Souza^1,2^, Hélio Vitoriano Nobre Júnior^1,2^; Livia Gurgel Do Amaral Valente Sá^1,2^; Bruno Coêlho Cavalcanti^2,4^; Islay Lima Magalhães^2^; José Roberto de Oliveira Ferreira^5^; Manoel Odorico de Moraes Filho^2,4^; João Batista de Andrade Neto^1,2,3^; Cecília Rocha da Silva^1,2^.

^1^Department of Clinical and Toxicological Analyses, Faculty of Pharmacy, Laboratory of Bioprospecting of Antimicrobial Molecules (LABIMAN), Federal University of Ceará, Fortaleza, CE, Brazil

^2^Nucleus for Drug Research and Development (NPDM), Federal University of Ceará, Fortaleza, CE, Brazil

^3^Christus University (UNICHRISTUS), Fortaleza, CE, Brazil

⁴Department of Physiology and Pharmacology, Faculty of Medicine, Federal University of Ceará, Fortaleza, CE, Brazil

⁵School of Medical Sciences, State University of Health Sciences of Alagoas (UNCISAL), Maceió, AL, Brazil.

* Corresponding author:

Telephone number: # 55 85 3366 8274

Fax: # 55 85 3366 8257

E-mail: labimanufc1@gmail.com.br

Online Resource 6 – Evaluation of the survival of mutant strains of *C. albicans*.

*p < 0.05 compared to control by ANOVA followed by Dunnett test. Data are presented as means ± S.D. for three independent experiments. DUL: duloxetina.

| Drug treatments |  | | *Candida*  *albicans* | | |
| --- | --- | --- | --- | --- | --- |
|  | WT | Cap1Δ | | Gpx3Δ |  |
| Negative Control | 100 ± 0.00 | 89.81 ± 2.40 | | 95.35 ± 4.47 |  |
| H₂O₂ | 82.49 ± 7.17 | 18.52 ± 3.73* | | 60.10 ± 4.37* |  |
| DUL 5μg/mL | 96.01 ± 4.52 | 64.16 ± 4.74* | | 87.27 ± 6.56* |  |
| DUL 10 μg/mL | 82.73 ± 2.11 | 35.21 ± 4.80* | | 68.35 ± 3.51* |  |
| DUL 30 μg/mL | 66.69 ± 6.16 | 11.56 ± 4.30* | | 43.88 ± 2.93* |  |
| DUL 60 μg/mL | 35.93 ± 5.30 | 5.66 ± 1.79* | | 23.01 ± 2.74* |  |
