# Supplementary material for: Duloxetine repositioning: Investigation of antifungal and antibiofilm activity against fluconazole-sensitive and resistant Candida Spp. strains
Source: Braz J Microbiol. 2026 Aug 3;57(1):226. doi: 10.1007/s42770-026-02038-z (PMC13433714; doi:10.1007/s42770-026-02038-z)
Supplement: Supplementary file 7 [file 42770_2026_2038_MOESM7_ESM.docx]

**Brazilian Journal of Microbiology**

Duloxetine Repositioning: Investigation of Antifungal and Antibiofilm Activity against Fluconazole-Sensitive and Resistant *Candida* Spp. Strains

Sarah Alves Barbosa^1,2^; Dávylla Rênnia Saldanha Pinheiro^1,2^; Leilson Carvalho de Oliveira^1,2^, Lara Elloyse de Almeida Moreira^1,2^, Vitória Pessoa Farias Cabral^1,2^, Daniel Sampaio Rodrigues^1,2^, Beatriz Oliveira de Souza^1,2^, Hélio Vitoriano Nobre Júnior^1,2^; Livia Gurgel Do Amaral Valente Sá^1,2^; Bruno Coêlho Cavalcanti^2,4^; Islay Lima Magalhães^2^; José Roberto de Oliveira Ferreira^5^; Manoel Odorico de Moraes Filho^2,4^; João Batista de Andrade Neto^1,2,3^; Cecília Rocha da Silva^1,2^.

^1^Department of Clinical and Toxicological Analyses, Faculty of Pharmacy, Laboratory of Bioprospecting of Antimicrobial Molecules (LABIMAN), Federal University of Ceará, Fortaleza, CE, Brazil

^2^Nucleus for Drug Research and Development (NPDM), Federal University of Ceará, Fortaleza, CE, Brazil

^3^Christus University (UNICHRISTUS), Fortaleza, CE, Brazil

⁴Department of Physiology and Pharmacology, Faculty of Medicine, Federal University of Ceará, Fortaleza, CE, Brazil

⁵School of Medical Sciences, State University of Health Sciences of Alagoas (UNCISAL), Maceió, AL, Brazil.

* Corresponding author:

Telephone number: # 55 85 3366 8274

Fax: # 55 85 3366 8257

E-mail: labimanufc1@gmail.com.br

Online Resource 7 – Effects of isolated DUL and DUL combined with AMB on biofilm formation against *Candida* spp. strains.

*p < 0.05 compared to control and ** p < 0.05 compared to AMB (MIC) by ANOVA followed by Tukey test. Data are presented as means ± S.D. for three independent experiments. AMB: anfotericina B; DUL: duloxetina.

| Drug treatments | *C. auris* | *C. albicans* | | *C. tropicalis* | *C. parapsilosis* |  |
| --- | --- | --- | --- | --- | --- | --- |
| DUL (MIC) | 35.00 ± 4.15* | 44.98 ± 0.95* | | 59.08 ± 5.10* | 50.12 ± 7.33* |  |
| DUL (2xMIC) | 2.17 ± 0.51* | 3.45 ± 0.18* | | 3.80 ± 0.22* | 47.89 ± 7.57* |  |
| DUL (4xMIC) | 1.53 ± 0.05* | 0.37 ± 0.14* | | 0.75 ± 0.15* | 16.61 ± 0.71* |  |
| DUL (8xMIC) | 0.60 ± 0.11* | 2.78 ± 0.27* | | 1.52 ± 0.40* | 59.77 ± 6.86* |  |
| AMB (MIC) | 97.36 ± 1.96 | 89.99 ± 3.49 | | 87.78 ± 3.59 | 103.64 ± 3.86 |  |
| AMB (2xMIC) | 72.42 ± 1.66 | 76.91 ± 2.58 | | 48.50 ± 1.94 | 80.95 ± 5.02 |  |
| DUL/AMB (MIC) | 89.41 ± 3.79 | 77.10 ± 2.46** | | 77.65 ± 2.77** | 95.06 ± 9.96 |  |
| DUL/AMB (2xMIC) | 75.47 ± 2.21** | 66.04 ± 0.82** | | 69.86 ± 2.48** | 68.24 ± 9.17 |  |
