# Supplementary material for: Duloxetine repositioning: Investigation of antifungal and antibiofilm activity against fluconazole-sensitive and resistant Candida Spp. strains
Source: Braz J Microbiol. 2026 Aug 3;57(1):226. doi: 10.1007/s42770-026-02038-z (PMC13433714; doi:10.1007/s42770-026-02038-z)
Supplement: Supplementary file 8 [file 42770_2026_2038_MOESM8_ESM.docx]

**Brazilian Journal of Microbiology**

Duloxetine Repositioning: Investigation of Antifungal and Antibiofilm Activity against Fluconazole-Sensitive and Resistant *Candida* Spp. Strains

Sarah Alves Barbosa^1,2^; Dávylla Rênnia Saldanha Pinheiro^1,2^; Leilson Carvalho de Oliveira^1,2^, Lara Elloyse de Almeida Moreira^1,2^, Vitória Pessoa Farias Cabral^1,2^, Daniel Sampaio Rodrigues^1,2^, Beatriz Oliveira de Souza^1,2^, Hélio Vitoriano Nobre Júnior^1,2^; Livia Gurgel Do Amaral Valente Sá^1,2^; Bruno Coêlho Cavalcanti^2,4^; Islay Lima Magalhães^2^; José Roberto de Oliveira Ferreira^5^; Manoel Odorico de Moraes Filho^2,4^; João Batista de Andrade Neto^1,2,3^; Cecília Rocha da Silva^1,2^.

^1^Department of Clinical and Toxicological Analyses, Faculty of Pharmacy, Laboratory of Bioprospecting of Antimicrobial Molecules (LABIMAN), Federal University of Ceará, Fortaleza, CE, Brazil

^2^Nucleus for Drug Research and Development (NPDM), Federal University of Ceará, Fortaleza, CE, Brazil

^3^Christus University (UNICHRISTUS), Fortaleza, CE, Brazil

⁴Department of Physiology and Pharmacology, Faculty of Medicine, Federal University of Ceará, Fortaleza, CE, Brazil

⁵School of Medical Sciences, State University of Health Sciences of Alagoas (UNCISAL), Maceió, AL, Brazil.

* Corresponding author:

Telephone number: # 55 85 3366 8274

Fax: # 55 85 3366 8257

E-mail: labimanufc1@gmail.com.br

Online Resource 8 – Effects of isolated DUL and DUL combined with AMB on mature biofilm against *Candida* spp. strains.

*p < 0.05 compared to control and ** p < 0.05 compared to AMB (MIC) by ANOVA followed by Tukey test. Data are presented as means ± S.D. for three independent experiments. FLC: fluconazol; AMB: anfotericina B; DUL: duloxetina.

| Drug treatments | *C. auris* | *C. albicans* | | *C. tropicalis* | *C. parapsilosis* |  |
| --- | --- | --- | --- | --- | --- | --- |
| DUL (MIC) | 85.09 ± 6.03* | 61.21 ± 2.17* | | 73.00 ± 2.46* | 40.48 ± 2.83* |  |
| DUL (2xMIC) | 45.53 ± 0.61* | 30.79 ± 0.84* | | 32.21 ± 1.91* | 28.23 ± 2.91* |  |
| DUL (4xMIC) | 12.50 ± 0.48* | 10.30 ± 1.07* | | 12.89 ± 0.67* | 13.07 ± 0.82* |  |
| DUL (8xMIC) | 6.71 ± 1.49* | 3.91 ± 0.57* | | 5.33 ± 0.20* | 8.83 ± 0.22* |  |
| AMB (MIC) | 98.69 ± 5.91 | 95.56 ± 6.78 | | 78.54 ± 1.90 | 98.22 ± 9.88 |  |
| AMB (2xMIC) | 94.47 ± 10.33 | 61.17 ± 10.99 | | 55.31 ± 4.67 | 92.73 ± 9.11 |  |
| DUL/AMB (MIC) | 102.91 ± 2.51 | 95.08 ± 4.47 | | 63.53 ± 1.95** | 95.73 ± 6.01 |  |
| DUL/AMB (2xMIC) | 99.35 ± 2.75 | 94.91 ± 4.31** | | 35.28 ± 3.51** | 87.08 ± 2.02 |  |
